# Small-molecule drugs development for Alzheimer's disease

**DOI:** 10.3389/fnagi.2022.1019412

**Published:** 2022-11-01

**Authors:** Weiwei Yao, Huihui Yang, Jinfei Yang

**Affiliations:** School of Health and Life Sciences, University of Health and Rehabilitation Sciences, Qingdao, China

**Keywords:** Alzheimer's disease, small molecule, Aβ, tau protein, therapeutic drugs monitoring

## Abstract

Alzheimer's disease (AD) is an irreversible, progressive neurodegenerative brain disorder with no effective therapeutic drugs currently. The complicated pathophysiology of AD is not well understood, although beta-amyloid (Aβ) cascade and hyperphosphorylated tau protein were regarded as the two main causes of AD. Other mechanisms, such as oxidative stress, deficiency of central cholinergic neurotransmitters, mitochondrial dysfunction, and inflammation, were also proposed and studied as targets in AD. This review aims to summarize the small-molecule drugs that were developed based on the pathogenesis and gives a deeper understanding of the AD. We hope that it could help scientists find new and better treatments to gradually conquer the problems related to AD in future.

## Graphical Abstract

Alzheimer's disease (AD) is an irreversible neurodegeneration brain disease, but there is no effective treatment of drugs. This review aims to summarize the recent development of small-molecule drugs for treating AD, so that researchers can understand the progress of AD drug research and development more deeply. We hope that this review can provide meaningful guidance for future AD drug development. 
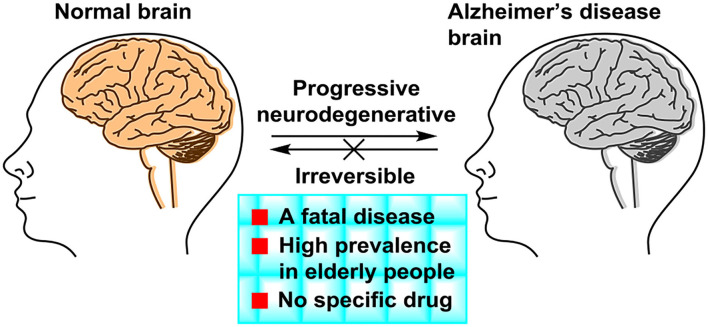

## Introduction

**Figure 1:**
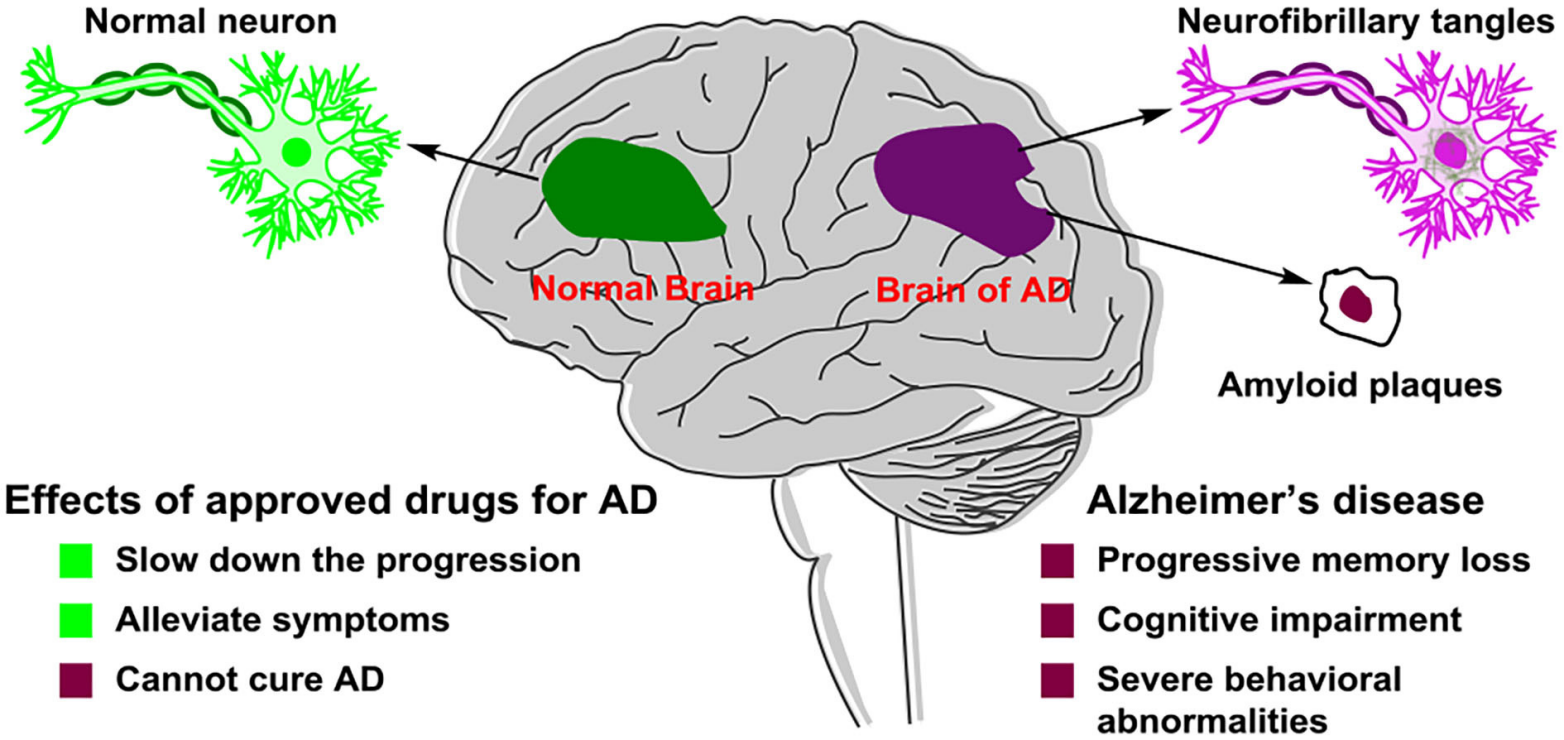
Pathological difference in neurons between a normal brain and an Alzheimer's disease (AD) patient's brain.

Alzheimer's disease (Lane et al., 2018) (AD) is a kind of progressive and irreversible neurodegenerative incurable brain disease among elderly people. As the global population ages, dementia has become one of the important health problems worldwide and AD is the most common form of dementia. AD accounts for 60% of all dementia cases. In developed countries, AD is considered as the third leading cause of death, following cardiovascular disease and cancer (Huang et al., 2016). The symptoms of AD (Figure 1) presented progressive memory loss, cognitive impairment, and severe behavioral abnormalities (Hu et al., 2017). The main two pathological hallmarks of AD are beta-amyloid (Aβ) plaques and phosphorylated tau-induced neurofibrillary tangles (Congdon and Sigurdsson, 2018). Owing to AD's complex pathophysiological characteristics, and complicated interactions with related genes and proteins, no effective drugs were available, which can obtain a long-term treatment effect of AD or even cure AD (Liu et al., 2017). The pathogenesis and mechanism of AD remain unclear, although five main types of pathogeneses, such as the beta-amyloid (Aβ) cascade (Mawuenyega et al., 2010), the hyperphosphorylated tau protein (Hanger et al., 2009), oxidative stress (Su et al., 2008), deficiency of central cholinergic neurotransmitters (Craig et al., 2011), and inflammation (Holmes, 2013), have been proposed. In the study of AD treatment, pathogenesis provides a basis for understanding and researching on AD.

Scientists have put enormous efforts in developing effective drugs to cure this fatal disease, although these studies are ending in failure currently. There are only five marketed drugs, including the inhibitors of acetylcholinesterase (AChEIs) such as Tacrine, Donepezil, Galantamine, Rivastigmine, and N-methyl-D-aspartate (NMDA) receptor antagonist Memantine (Reitz et al., 2011), as approved by the Food and Drug Administration (FDA) to treat AD (Guzior et al., 2015). However, although these marketed drugs can slow down the progression of the disease and alleviate symptoms, they cannot ultimately cure the disease. Therefore, there is a critical need to develop new and effective drugs that can prevent, delay, or slow down the progression of AD. The study of AD treatment is mainly based on the pathological changes. To offer some help to the development of AD therapeutic drugs in future, we write this review to summarize some representative AD-treatment small-molecule drugs that entered clinical trials mostly.

## Small molecules

### Cholinergic inhibitors

**Figure 2:**
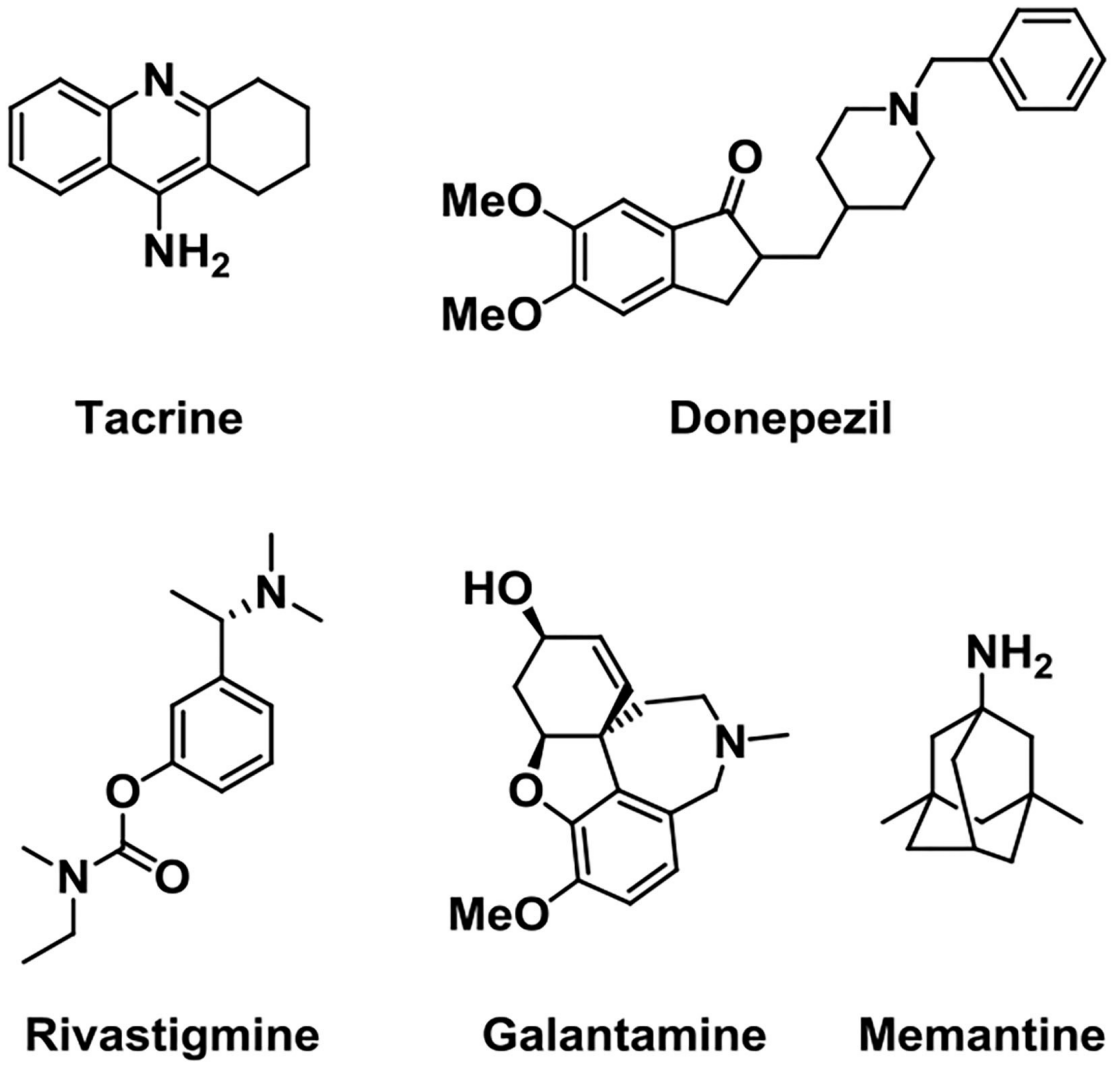
Structures of five drugs approved by the Food and Drug Administration (FDA) in the treatment of Alzheimer's disease (AD).

In 1993, FDA approved the use of the cholinesterase inhibitor such as Tacrine to treat mild-to-moderate AD patients first (Relman, 1991), and subsequently approved three similar drugs such as Donepezil, Galantamine, and Rivastigmine (Figure 2). However, other cholinesterase inhibitors, such as velnacrine, physostigmine, eptastigmine, and metrifonate reported, did not get approval (Becker et al., 2008). As an NMDA receptor antagonist, Memantine was approved as a moderate-to-severe AD drug, which acted on the glutamatergic system (Reisberg et al., 2003; Olivares et al., 2012).

Other kinds of small-molecule agents that targeted AChE have also been developed. Huperzine A is a sesquiterpene alkaloid derived from the fern Huperzine, and it was found to cause a reversible inhibition of AChE with high efficiency and low toxicity (Damar et al., 2016). Huperzine A can increase the levels of acetylcholine in the body, improve the functions of brain and nerve, and cause an improvement in cognitive function (Yang et al., 2013). It has been discovered that Huperzine A showed a promising effect on the treatment of AD, and it was listed in China for the treatment of AD in 1995. Through structural modification, the pro-drug ZT-1 derived from Huperzine A presented a better selectivity and higher therapeutic index (Jia et al., 2013). This optimized structure presents a rapid absorption and a wide distribution in humans.

Physostigmine is a potent parasympathomimetic alkaloid and reversible acetylcholinesterase inhibitor which can provide a rapid reversal of the anticholinergic toxicity (Califf, 2018). The narrow therapeutic window as well as side effects of this drug limit its treatment in AD (Coelho and Birks, 2001; Batiha et al., 2020). Then, scientists were devoted to developing a derivative of Physostigmine with a better effect over the years, and (–)-phenserine presented a cognitive improvement. Besides, the study showed that (–)-phenserine can reduce the translation of amyloid precursor protein (APP) to reduce Aβ concentrations, indicating that analogs of (–)-phenserine may be a potential drug for AD (Winblad et al., 2010).

An analog of galantamine was developed. Memogain (Gln-1062) is an inactive pro-drug of galantamine, and it works by enzymatically cleaving galantamine, then it will regain its pharmacological activity as a cholinergic enhancer (Maelicke et al., 2010). It has been reported that Memogain has more than 15-fold higher bioavailability in the brain than the same doses of galantamine, owing to its enhanced hydrophobicity. According to preclinical data, the nasal inhalation use of Memogain can avoid gastrointestinal side effects and present a higher potency in enhancing cognition (Bhattacharya et al., 2015).

Ladostigil (TV-3326) is an orally active potential treatment drug for AD (Weinreb et al., 2008). In the structure of Ladostigil, a carbamate cholinesterase inhibitory (ChEI) moiety of rivastigmine (Weinstock et al., 1994) was contained in the aminoindan structure of the monoamine oxidase (MAO)-B inhibitor. It is a dual inhibitor of cholinesterase and brain-selective monoamine oxidases (MAO) A and B, with IC_50_s (half-maximal inhibitory concentrations) of 37.1 and 31.8 μM for MAO-B and AChE, respectively. In addition, ladostigil exhibits neuroprotective, antioxidant, and anti-inflammatory activities including the regulation of amyloid precursor protein (APP) processing (Yogev-Falach et al., 2002; Bar-Am et al., 2004), activation of protein kinase C, and mitogen-activated protein kinase signaling pathways.

NGX267 (AF267B) is a functionally selective partial M1 agonist that offers prospects for treating memory and cognitive disturbances (Caccamo et al., 2006; Ivanova and Murphy, 2009). The experiment on AD transgenic mice proved that NGX267 represents an effective peripherally administered drug to attenuate the major hallmarks of AD and to reverse deficits in cognition, like the reduction of the Aβ and tau pathologies in the hippocampus and cortex.

**Figure 3:**
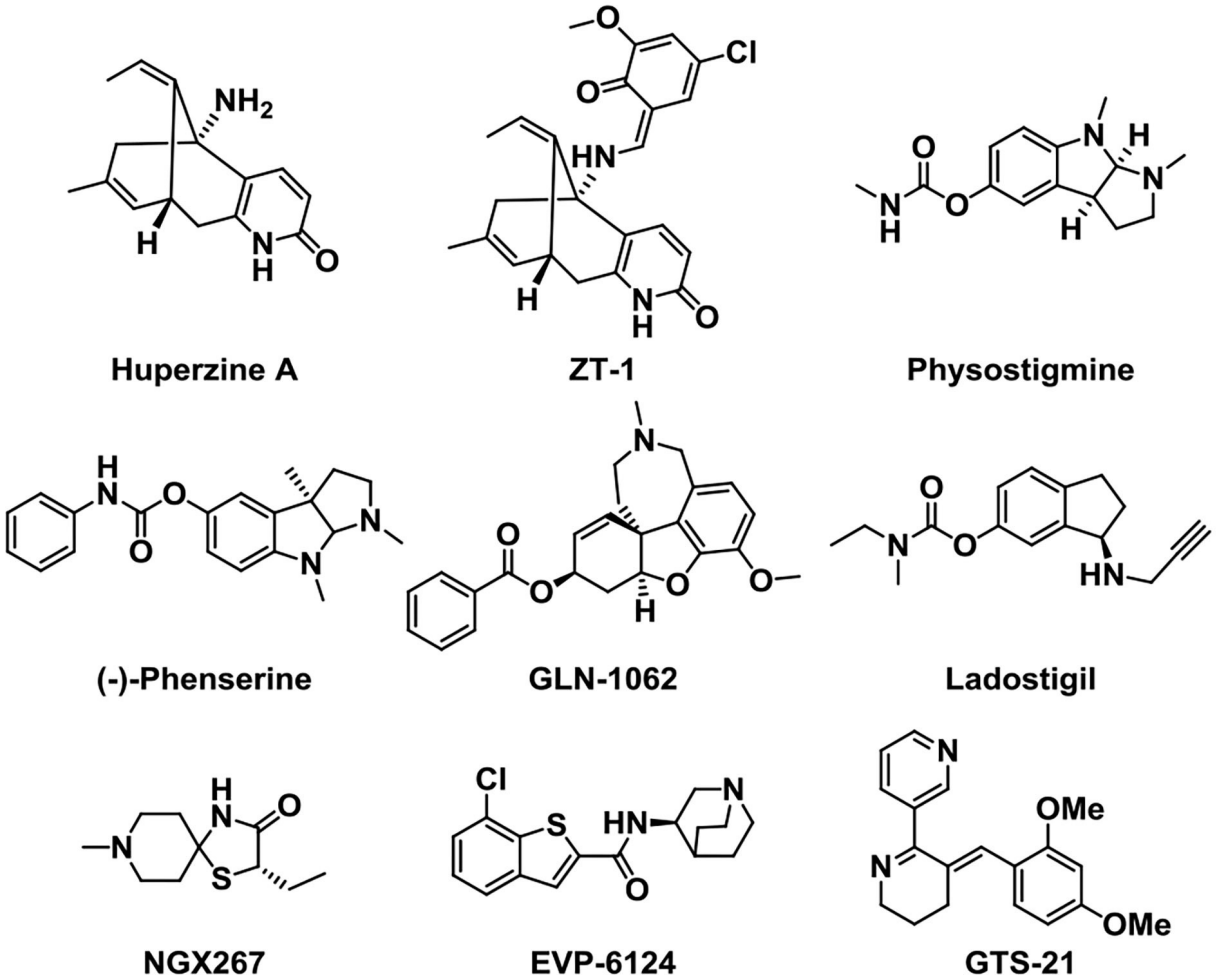
Structures of cholinergic inhibitors in the study.

EVP-6124 is a novel partial agonist of α7 neuronal nicotinic acetylcholine receptors (nAChRs) (Lendvai et al., 2013) with good brain penetration and an adequate exposure time and presents high affinity *in vivo* and *in vitro* (Prickaerts et al., 2012). It activates the α7 nicotinic acetylcholine receptor at low nanomolar brain concentrations and improves the memory performance in rats. Although donepezil at 0.1 mg/kg, po (oral) or EVP-6124 at 0.03 mg/kg, po did not improve memory, co-administration of these doses can restore memory. Based on the relevant clinical trials and proposed mechanism, EVP-6124 combined with cholinesterase inhibitors probably provides a good therapeutic strategy in cognitive impairment (Deardorff et al., 2015). In addition, GTS-21 is also a selective α7 nicotinic acetylcholine receptor (nAChR) agonist with the anti-inflammatory and cognition-enhancing function (Zawieja et al., 2012; Garg and Loring, 2019) (Figure 3).

### Equations decreasing Aβ production

#### BACE1 inhibitors

**Figure 4:**
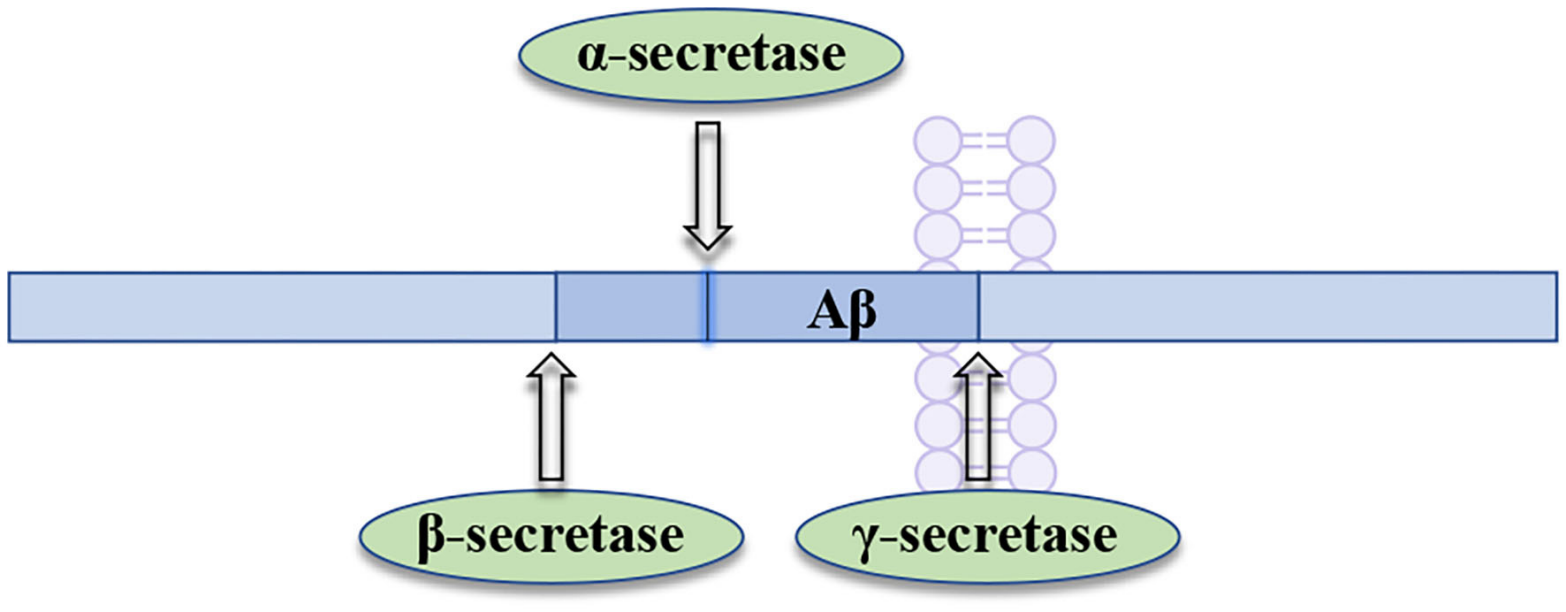
Processing of amyloid precursor protein (APP) by β-secretase and **γ-**secretase.

BACE1 (the beta-site APP-cleaving enzyme 1), acting as a membrane-anchored aspartic acid protease, is an enzyme that promotes the generation of neurotoxic Aβ in brain (Hampel et al., 2021). BACE1 has been widely pursued as an AD drug target, owing to its critical role in the production of amyloid-beta (Aβ) (Mullard, 2017). BACE1 inhibition can prevent the formation of Aβ at the very beginning of APP processing (Figure 4), slowing down the progression of AD by inhibiting Aβ formation at an early stage. Therefore, the therapeutic potential of BACE1 inhibitors is currently tested in the clinical trials for AD treatment (Piton et al., 2018).

**Figure 5:**
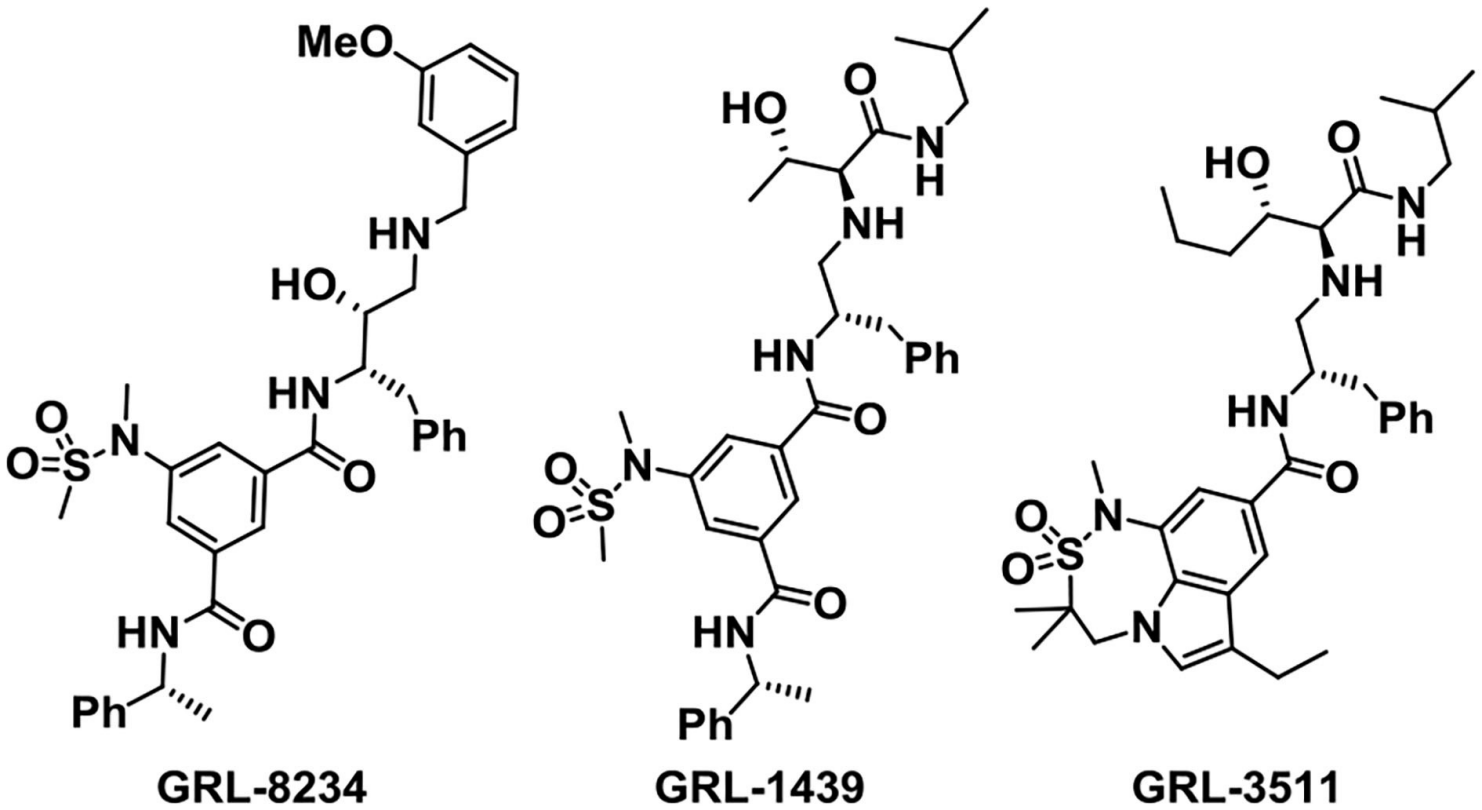
Structures of GRL-8234, GRL-1439, and GRL-3511.

The Ghosh group has designed and synthesized plenty of small-molecule BACE1 inhibitors according to the structure-based design strategy. Among the molecules, GRL-8234 (Figure 5) exhibited excellent properties with an IC_50_ of 1.0 nM. Treatment with GRL-8234 could reduce the interstitial fluid Aβ40 in Tg2576 mice and rescue age-related cognitive decline (Chang et al., 2011). Exceedingly potent and selective BACE1 inhibitors like GRL-1439 and GRL-3511 were also developed and the X-ray crystal structure binding BACE1 was characterized to explore the mechanisms (Ghosh and Osswald, 2014). The challenges of developing BACE1 inhibitors are selectivity and brain penetration. Among the BACE1 drug candidates developed by the Ghosh group, clinical trials of about 13 compounds were carried out.

**Figure 6:**
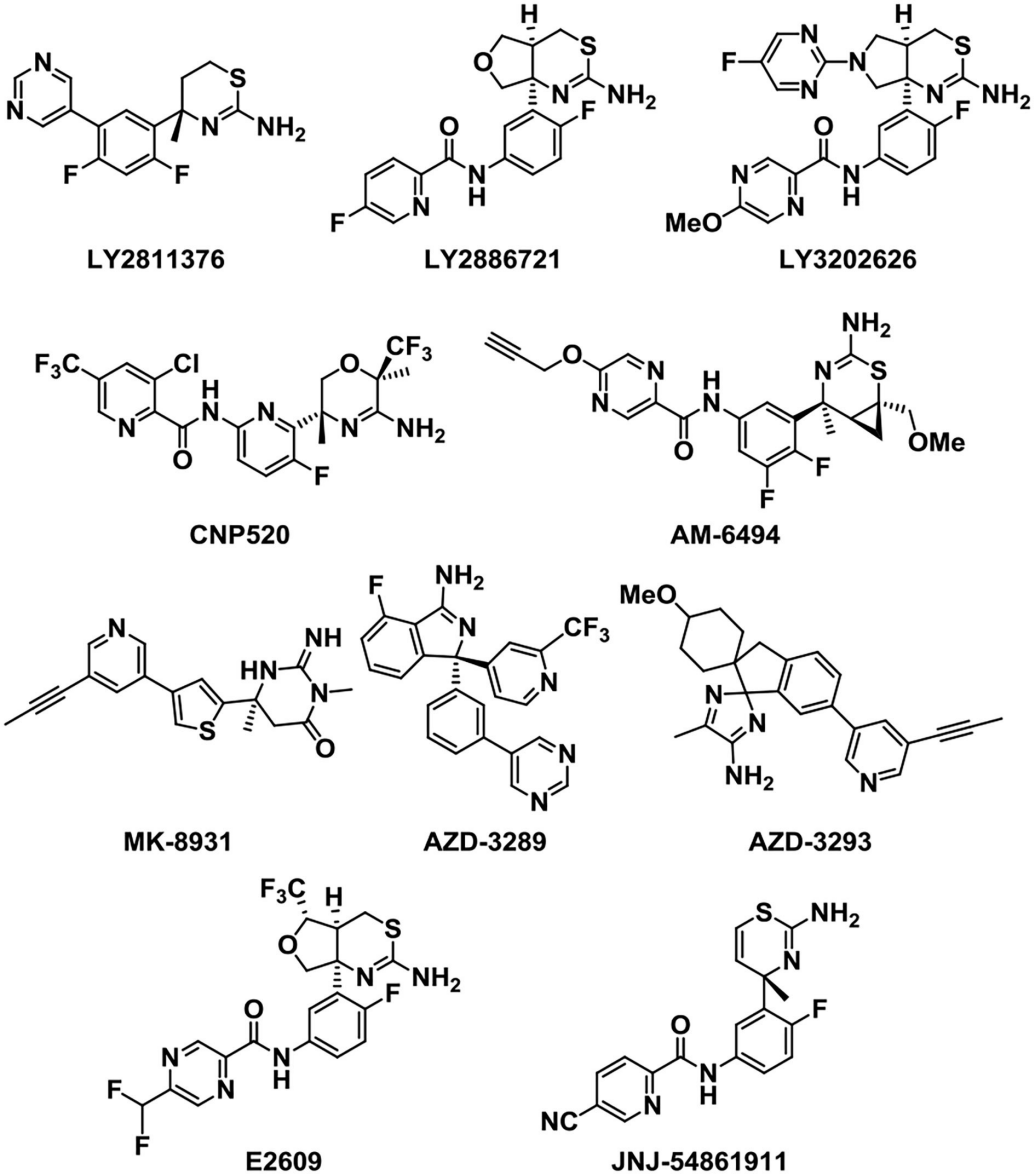
Structures of the beta-site APP cleaving enzyme 1 (BACE1) inhibitors in study.

Because of the vital role of BACE1 in the generation of Aβ, BACE1 inhibitors have attracted many scientists to the AD treatment area. Next, we will introduce some potent BACE1 inhibitors that entered clinical trials (Figure 6). LY2811376 is the first reported oral BACE1 inhibitor that leads to profound Aβ-lowering effects in nonclinical animal models and healthy volunteers (May et al., 2011). However, it was terminated in Phase I due to a nonclinical retinal toxicity upon longer term dosing. Then, LY2886721, the second-generation orally available BACE1 inhibitor, reached phase 2 clinical trials in AD (May et al., 2015). It has a high selectivity against key off-target proteases. But the development was stopped because liver enzymes were abnormally elevated. In order to reduce the side effect of liver injury, a more potent BACE1 inhibitor capable of achieving high BACE1 inhibition at lower clinical doses was developed (McKinzie et al., 2021). The third-generation BACE1 inhibitor, LY3202626, a small molecule which was proved to reduce amyloid-β (Aβ)_1-40_ and Aβ_1-42_ concentrations in plasma and cerebrospinal fluid. It characterized a highly potent, CNS penetrant, and low-dose BACE1 inhibitor (Lo et al., 2021).

Verubecestat (MK-8931), developed by the pharmaceutical company Merck, is a potent, selective, structurally unique BACE1 inhibitor that can reduce the plasma, cerebrospinal fluid (CSF) (Forman et al., 2012), and brain concentrations of Aβ40, Aβ42, and soluble APP beta protein (sAPPβ) (a direct product of the BACE1 enzymatic activity) in both healthy human subjects and AD patients (Forman et al., 2013). Research shows that MK-8931 presents few adverse effects compared to previously reported BACE inhibitions, and single and multiple doses were generally well tolerated (Kennedy et al., 2016). The human data are fit to an amyloid pathway model and provide a direction for the subsequent clinical trials. MK-8931 is the first BACE1 inhibitor to reach phase III clinical trials (Kennedy et al., 2016).

Umibecestat (CNP-520) is an orally available small-molecule BACE1 inhibitor which attained phase II/III clinical trials along with MK-8931. However, the clinical trials were discontinued lately in February 2018, as participants displayed worsened cognitive functions (Neumann et al., 2018). Recently, AM-6494 was discovered as a novel potent and orally effective BACE1 inhibitor that advanced to preclinical development (Pettus et al., 2020). AM-6494 showed a higher selectivity and inhibitory potency for BACE1 than CNP-520 (Ugbaja et al., 2020).

In addition, E2609 and JNJ-54861911 were other two BACE1 inhibitors that reached phase III clinical trials. E2609 is an orally available BACE1 inhibitor and can highly reduce the Aβ levels of the CSF and plasma in rodents in a dose-dependent manner (Lai et al., 2012; Bernier et al., 2013). JNJ-54861911 has proved to be very successful in reducing Aβ levels, which can reach up to 95% reduction after administration in healthy volunteers (Novak et al., 2020). However, the clinical trials were discontinued in phase II/III owing to the safety problems (Patel et al., 2022). Other analogs that acted as BACE1 inhibitors were also developed in the following study.

As a potent and selective BACE1 inhibitor, AZD-3839 can effectively reduce the levels of Aβ in brain, CSF, and plasma in several preclinical species in a dose- and time-dependent manner (Jeppsson et al., 2012). Although AZD-3839 had advanced to phase 1 clinical trials in May 2011 in the UK, the clinical trials were discontinued in December 2012 (Swahn et al., 2012). AZD3293 is another potent, highly permeable, orally active BACE1 inhibitor which has a good blood–brain barrier (BBB) penetration. AZD3293 presented significant dose- and time-dependent reductions in plasma, CSF, and brain concentrations of Aβ40, Aβ42, and sAβPPβ and a slow off-rate, and an excellent *in vivo* efficacy with a prolonged on-target effect (Eketjäll et al., 2016). It has entered the clinical development as a promising disease-modifying treatment for AD.

To date, none of the BACE1 inhibitors succeeded in slowing or reversing the progression of AD in clinical trials, owing to the adverse effects or the invalid nature in promoting the cognitive ability. These results might encourage researchers to reconsider whether BACE1 inhibitors are effective therapeutic agents in AD treatment.

#### γ-secretase inhibitors and modulators

**Figure 7:**
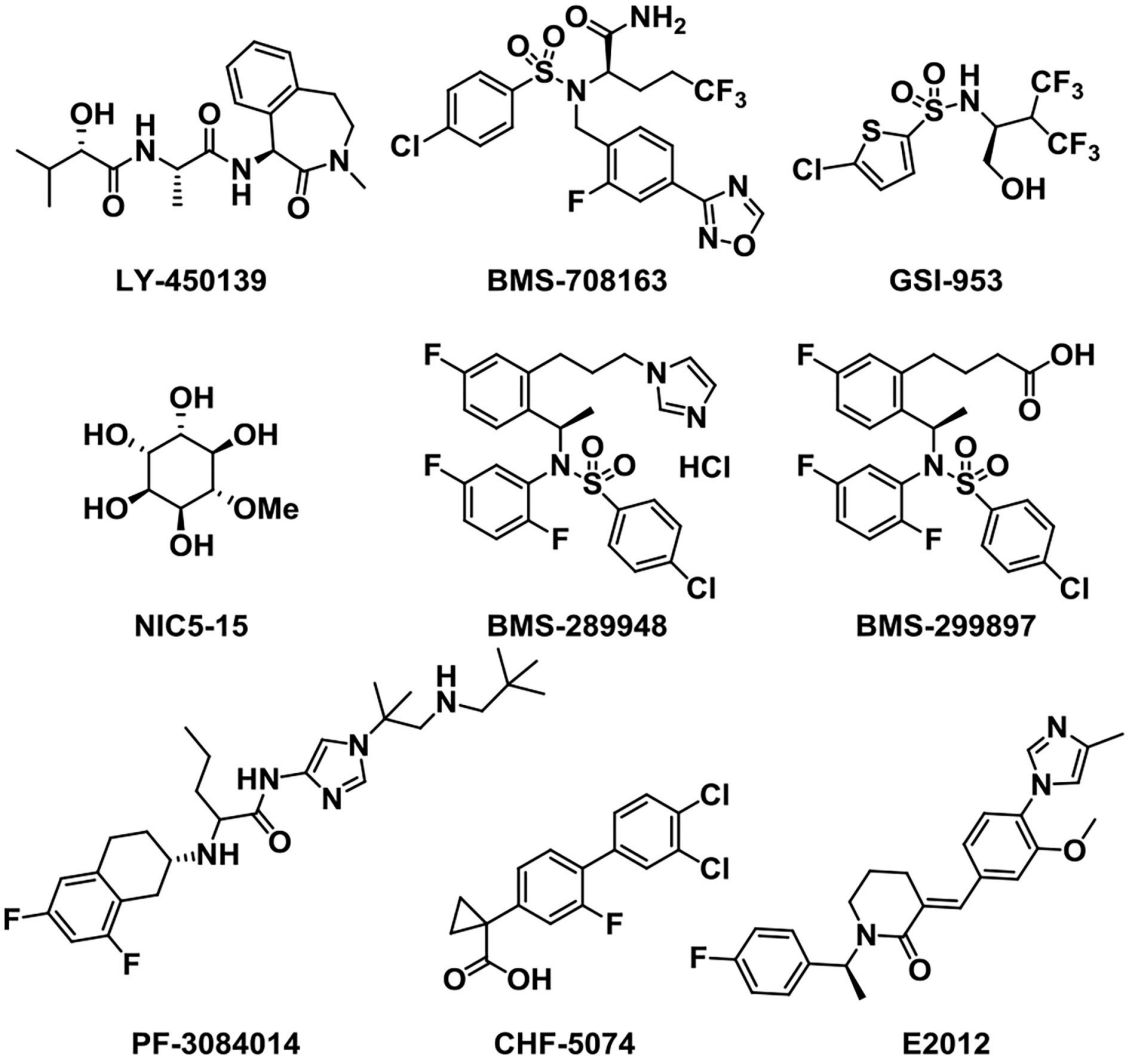
Structures of γ-secretase inhibitors and modulators.

As well as BACE1 inhibitors, the inhibition of γ-secretase can also reduce Aβ production and decrease the accumulation of Aβ oligomers and plaques, providing an effective treatment for AD (Wolfe, 2008; Golde et al., 2010). Therefore, the development of γ-secretase inhibitors and modulators (Figure 7) represents an attractive therapeutic opportunity for AD (Yang et al., 2021). Semagacestat (LY-450139), developed and named by Eli Lily (Henley et al., 2009), was the first γ-secretase inhibitor (GSI) that entered phase III clinical research. The clinical trials were stopped owing to severe side effects like the cleavage inhibition of other substrates such as Notch signaling proteins and other cell surface receptors (Doody et al., 2015; Aster et al., 2017). To solve this problem, the notch-sparing GSIs were developed such as Avagacestat (BMS-708163), which preferentially inhibited the cleavage of APP over Notch (Gillman et al., 2010). But, BMS-708163 did not show an obvious efficacy in Phase II trials and high doses produced toxicity (Coric et al., 2012, 2015). Begacestat (GSI-953) (Martone et al., 2009) is another GSI which can selectively inhibit the cleavage of APP over Notch, showing a promise in recent Phase I clinical trials (Hopkins, 2012). In healthy human volunteers, the oral administration of a single dose produces dose-dependent changes in plasma Aβ levels, proving the pharmacodynamic activity of GSI-953 in humans. NIC5-15 is a natural product found in pine bark and many foodstuffs such as soy and carob (Grossman et al., 2009). In transgenic mice, it is proved to diminish the production of Aβ1-42 *via* notch-sparing γ-secretase inhibition. NIC5-15 has a safe and effectual treatment in improving cognitive function in mild-to-moderate AD patients (Pasinetti et al., 2009).

PF-3084014 is a potent γ-secretase inhibitor that reduces Aβ production with an IC_50_ of 1.2 nM (whole-cell assay) *in vitro*. It showed a dose-dependent reduction in brain, CSF, and plasma Aβ in Tg2576 mice as measured (Lanz et al., 2010). In the family of GSIs, BMS-289948 and BMS-299897 are two orally active γ-secretase inhibitors. They can markedly reduce plasma and brain Aβ level in a time- and dose-dependent manner (Anderson et al., 2005).

In addition to GSIs, γ-secretase modulators (GSMs) have also been acting as attractive small molecules for alleviating the symptoms of AD (Kounnas et al., 2010; Crump et al., 2013). GSMs may promote further cleavage of Aβ42, thus reducing the amount of aggregation-prone Aβ peptides (Pozdnyakov et al., 2013). In transgenic mice of the AD model, treatment with CHF5074 can markedly reduce brain Aβ burden without any histological peripheral Notch-mediated toxicity (Imbimbo et al., 2009). CHF5074 shows a dose-dependent manner and well tolerated and safety in mild-to-moderate patients (Ross et al., 2013). This γ-secretase modulator is a promising therapeutic agent for AD. Besides, the small molecule E2012 is a widely used classic member of the heterocyclic GSM family without affecting Notch processing, aiming at AD by reduction of Aβ40 and Aβ42 in a dose-dependent manner (Nakano-Ito et al., 2013).

### Preventing Aβ aggregation

**Figure 8:**
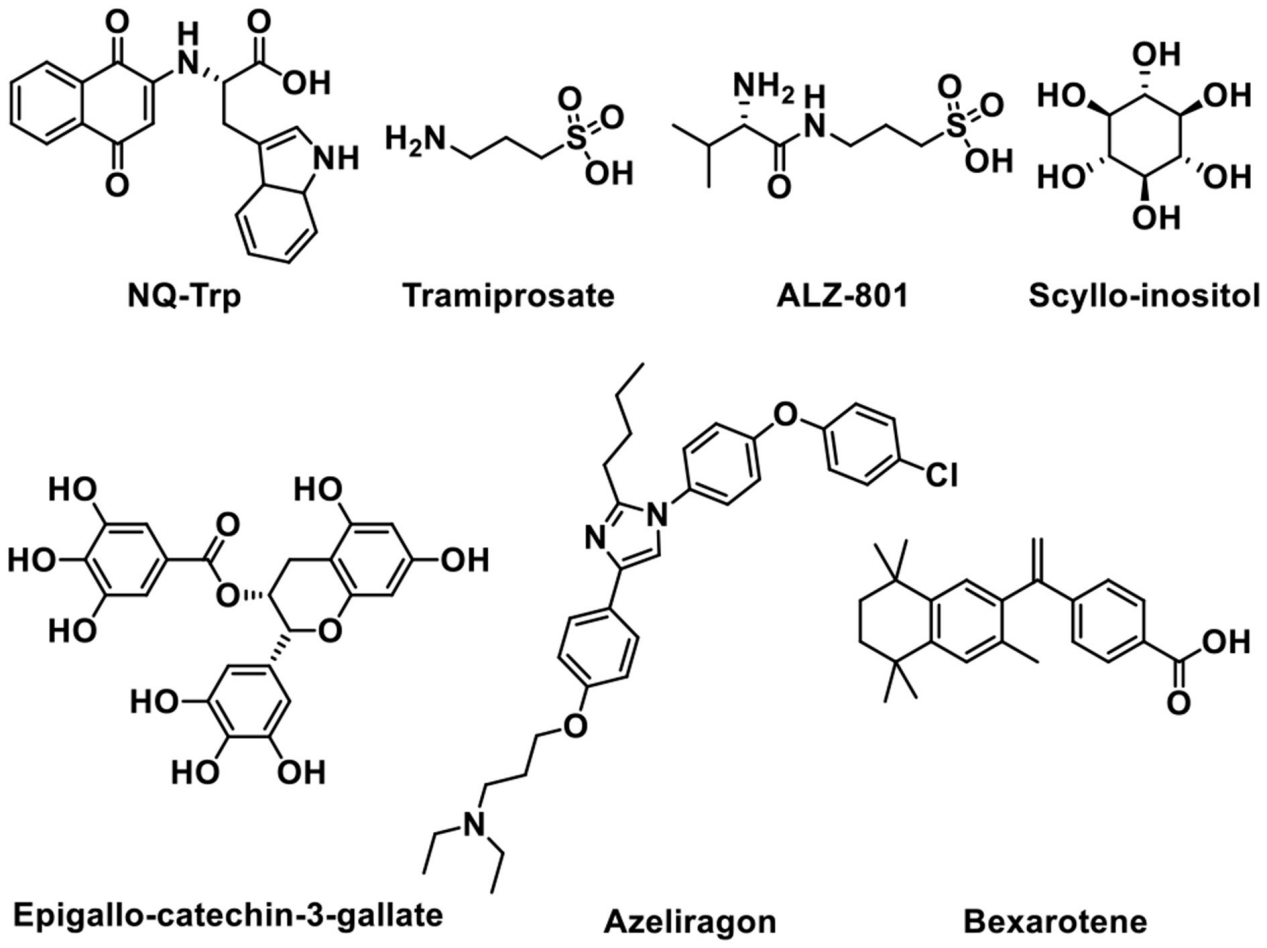
Structures of molecules in preventing amyloid beta (Aβ) aggregation.

The aggregation of monomeric peptide Aβ into amyloid fibrils is one of the features and pathogenies of AD. Oligomers, the aggregation intermediates, are toxic and cause neuronal cell death (Haass and Selkoe, 2007; Glabe, 2008). Therefore, inhibiting the formation of oligomers and aggregates is widely studied as a promising therapeutic treatment for Alzheimer's disease (Hardy and Selkoe, 2002). Next, some representative small-molecule agents that can prevent Aβ aggregation will be introduced (Figure 8).

Tramiprosate is an orally administered small molecule that binds to soluble amyloid, thus inhibiting amyloid aggregation in the brain (Aisen et al., 2007). Preclinical and clinical studies showed that the oral treatment of tramiprosate could reduce oligomeric and fibrillar (plaque) amyloid, diminish hippocampal atrophy, improve cholinergic transmission, and stabilize cognition (Manzano et al., 2020). Although tramiprosate is safe and well tolerated, the clinical trials were terminated in Phase III trial owing to the failure in demonstrating efficacy (Aisen et al., 2011; Herrmann et al., 2011). ALZ-801 is an oral valine-conjugated pro-drug of tramiprosate, providing significantly improved pharmacokinetic variability and gastrointestinal tolerance (Hey et al., 2018b), inhibiting the formation of amyloid oligomers without plaque interaction. 3-Sulfopropanoic acid (3-SPA), the main metabolite of tramiprosate and ALZ-801, is an endogenous molecule that is present in the brain of patients with AD (Hey et al., 2018a). It has an anti-Aβ aggregation activity *in vitro* with an efficacy comparable to tramiprosate. ALZ-801 showed excellent oral safety and tolerability in healthy adults and elderly volunteers with significantly improved pharmacokinetic characteristics over oral tramiprosate (Tolar et al., 2020). Therefore, ALZ-801 may be an advanced and markedly improved clinical candidate drug for the treatment of AD (Tolar et al., 2019).

*Scyllo*-inositol, one of the nine structural isomers of inositol (Fenili et al., 2007), can reduce amyloid toxicity and regulate myo-inositol levels to improve cognitive function in patients (Salloway et al., 2011). When taken orally, *Scyllo*-inositol is able to reach the brain. *Scyllo*-inositol showed amyloid- and myo-inositol-lowering effects in CSF and brain in AD patients (Ramp et al., 2021). A Phase II clinical research provided acceptable safety, showing that *Scyllo*-inositol might be a promising therapeutic agent for AD (Ma et al., 2012). Another natural product named Epigallo-catechin-3-gallate (EGCG), a major polyphenol component of green tea existed in the leaves of *Camellia sinensis*, can cross the BBB, showing antioxidant and neuroprotective functions (Mandel et al., 2008). It can prevent the formation of Aβ toxic oligomers through binding to unfolded peptide. In addition, EGCG can protect brains against aluminum chloride (AlCl_3_) toxicity and promote the defense of mitochondrial and cholinergic synaptic functions (Jayasena et al., 2013; Ortiz-López et al., 2016).

It is reported that naphthoquinone tryptophan (NQ-Trp) works by inhibiting Aβ aggregation and rescues cells from Aβ toxicity (Berthoumieu et al., 2015). However, NQ-Trp presents a relatively weak inhibitor, attributed to no specific “binding site”-type interaction with mono- and dimeric Aβ.

Azeliragon (TTP488 or PF-04494700) is an advanced glycation end-products (RAGE) inhibitor investigated in clinical trials (Sabbagh et al., 2011; Burstein et al., 2014). It inhibits the interactions between RAGE and its ligands, including Aβ_1-42_, HMGB1 (high mobility group box 1), S100B, and CML (chronic myeloid leukemia) (Cummings et al., 2020). Phase II clinical trial data of Azeliragon proved that it could delay the time period to cognitive deterioration in mild AD patients (Burstein et al., 2016, 2018). Unfortunately, owing to the toxicity, the dosage in phase III clinical trial was limited to 5 mg/day without obtaining any expected results (Xie et al., 2021).

In addition, Bexarotene has a high BBB permeability and it can be used in the treatment of AD as a potential therapeutic agent (Cramer et al., 2012). Bexarotene can improve memory and cognitive activity by binding to peroxisome proliferator-activated receptor gamma (PPAR-γ) and retinoid X receptors (RXRs), increasing apolipoprotein E (ApoE) expression and Aβ clearance (Tai et al., 2014).

### Tau aggregation inhibitors

Paired helical filaments (PHFs) and neurofibrillary tangles (NFTs), composed of various tau species, are usually observed in AD patients' brains (Bejanin et al., 2017). Therefore, tau protein is regarded as a promising target for AD. Tau pathology has a greater impact on the cognitive decline progression of AD patients than amyloid β (Shafiei et al., 2017). Abnormal phosphorylation of tau followed by dissociation from microtubules has been considered as the key event that initiates tau pathologies in AD. It is reported that the inhibition of toxic tau oligomers was considered an effective therapeutic approach for AD (Cárdenas-Aguayo et al., 2014).

**Figure 9:**
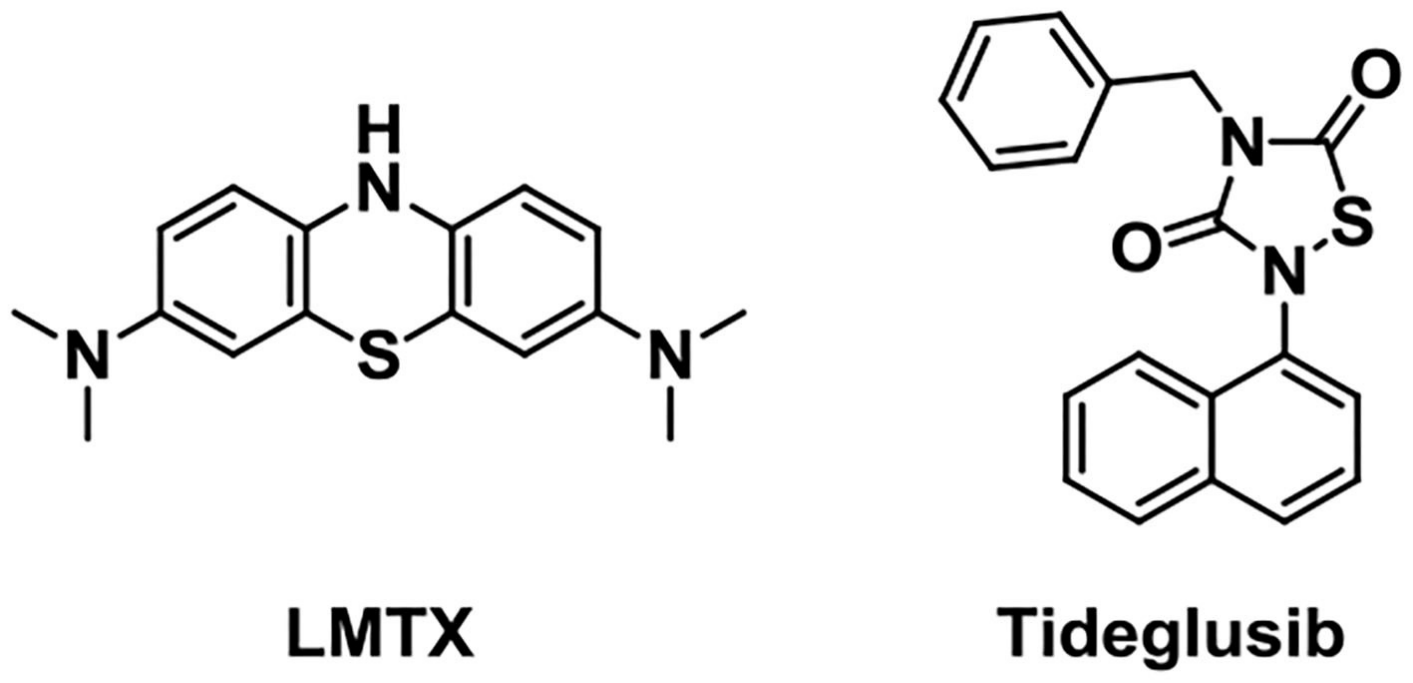
Structures of tau aggregation inhibitors.

Glycogen synthase kinase 3 (GSK-3), one of the multifunctional serine/threonine kinases, is a central mediator molecule of harmful inflammatory mechanisms relevant to AD. It contains two isoforms, GSK3α and GSK3β. GSK3β is abundant in the brain and it is a key target that regulates tau phosphorylation (Morales-Garcia et al., 2012). Tideglusib (Figure 9, NP-031112), an in-house non-ATP (adenosine triphosphate) competitive GSK-3β inhibitor, is under Phase IIa and IIb clinical trials. Preclinical studies showed that it could reduce tau phosphorylation, amyloid deposition, neuron loss, and gliosis (Luna-Medina et al., 2007; Pradeepkiran and Reddy, 2021).

Preventing tau interaction and neurofibrillary tangle accumulation could be a promising treatment for AD. LMTX (TRx0237), a stable reduced form of the methylthioninium (MT) moiety, has been extensively studied as a tau aggregation inhibitor (Gauthier et al., 2016). It has been found to show potential efficacy as monotherapy during the test in Phase 2 and 3 clinical trials in AD (Lee et al., 2019).

### Other targeted drugs

**Figure 10:**
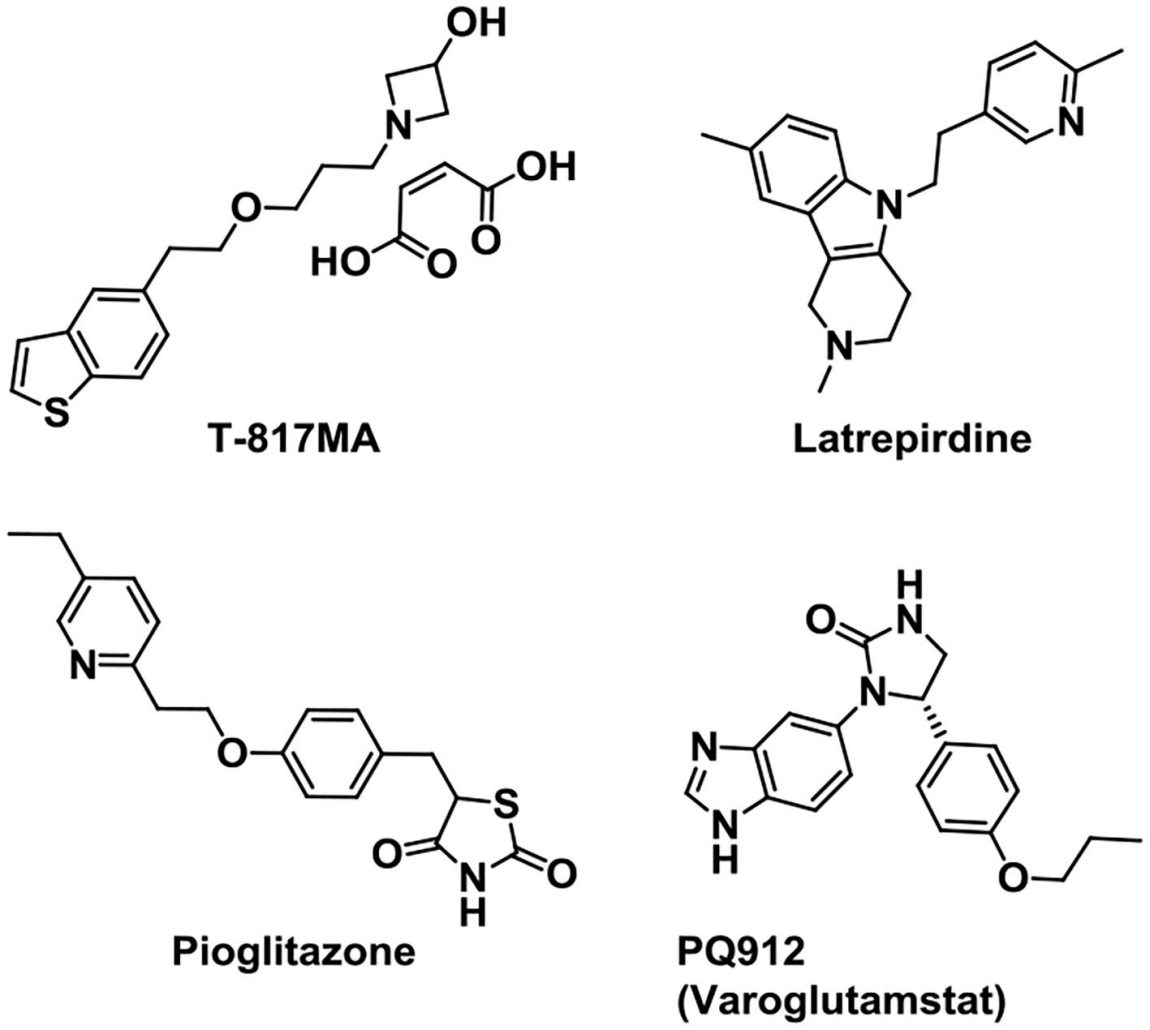
Structures of other targeted drugs.

Neurotrophins can regulate the generation, survival, proliferation, differentiation, and death of neurons in the nervous system. They refer to a family of proteins, nerve growth factor (NGF), brain-derived neurotrophic factor (BDNF), neurotrophin-3 (NT-3), and neurotrophin-4 (NT-4) (Sahay et al., 2017). Based on the above, some drugs were synthesized targeted to neurotrophins in AD treatment (Figure 10). As a σ-1 R agonist, T-817MA is a neuroprotective agent which could improve the motor and cognitive impairments in neuronal degeneration transgenic mice (Nguyen et al., 2007; Fukushima et al., 2011). It shows a potential for AD treatment in preclinical studies. Additionally, it can also provide protection against mitochondrial damage (Fukushima et al., 2006) and give protection against Aβ toxicity (Kimura et al., 2009). However, the early results in clinical trials showed little improvement (Schneider et al., 2019) and further clinical trials are ongoing.

Mitochondrial dysfunction is considered as one of the most emerging pathological processes in neurological disorders. And mitochondrial dysfunction is regarded as the core pathological process in AD (Wu et al., 2019). In addition, it has been reported that synaptic damage and mitochondrial dysfunction are the early causes in AD progression (Reddy et al., 2010). Therefore, targeting mitochondrial dysfunction to prevent the AD progression presents a promising therapeutic strategy. Latrepirdine (sold as Dimebon) is an orally available nonselective antihistamine developed in Russia since 1983 (Matveeva, 1983), and later it had been used in the treatment for AD by Medivation, Inc. and Pfizer (Bachurin et al., 2001). It is reported that treatment with latrepirdine can affect cellular functions like multireceptor activity, mitochondrial function, calcium influx, and intracellular catabolic pathways (Bharadwaj et al., 2013). Although the double-blind, placebo-controlled phase II trial in 2008 presented an improved clinical outcome in mild-to-moderate AD, latrepirdine failed to show efficacy in the cognition improvement in phase III trials (Chau et al., 2015).

Pioglitazone (AD4833), a kind of PPAR-γ agonist with high affinity, is an insulin-sensitizing agent used for type 2 diabetes (Verschuren et al., 2014). It forms a heterodimer with RXR and affects the transcription of genes relevant to glucose and lipid metabolism and inflammation reduction (Bogacka et al., 2004; Ko et al., 2008). Due to its positive effects on cerebral glucose, lipid metabolism, and inflammation, pioglitazone is also useful in the treatment of AD. Phase II study (Geldmacher et al., 2011; Saunders et al., 2021) in AD and previous trial showed that pioglitazone is safe and well tolerated. The Phase III trial named “TOMORROW” study, treatment of pioglitazone to the high-risk AD asymptomatic participants, showed a delayed effect on cognitive impairment (Burns et al., 2021).

Glutaminyl cyclase (QC) inhibitor PQ912 (Varoglutamstat) showed a robust therapeutic effect in transgenic mouse models of AD. Data proved that brain QC is a druggable target and QC inhibitor PQ912 can reduce the formation of pyroglutamyl-Aβ (Hoffmann et al., 2017). The phase 1 and phase 2a studies in healthy volunteers showed that PQ912 was safe and well tolerated (Lues et al., 2015; Scheltens et al., 2018). It is discovered that a combination of the QC inhibitor PQ912 and the murine monoclonal antibody PBD-C06 (m6) gives additive effects on brain Aβ pathology in transgenic mice (Hoffmann et al., 2021). Combining two treatment strategies might provide new ways for the AD treatment.

## Conclusion and outlook

In summary, the main research works focused on decreasing Aβ, which is regarded as a key player in AD development and progression. However, none of the drugs explored originating from Aβ hypothesis has passed clinical trials. Although the corresponding treatment can decrease the levels of Aβ in blood and CSF and clear Aβ deposits in the brain, cognitive functions did not improve. Other drugs targeting subpathologies, like neurofibrillary tangles comprising tau protein, neuroinflammation, oxidative stress, and so on, did not get satisfactory results.

Attributed to the complex biological machinery with complicated genes and proteins, AD presents a challenging problem in the study. Although current AD treatments can reduce and temporarily slow down the symptoms of AD, they cannot stop the progression of brain damage. The five approved prescription drugs by FDA do not show a good efficacy and tolerability in a wide range of patients, especially for the severe and advanced cases of AD. To date, there is no cure for this fatal disease. It is vital to design and develop a new treatment to terminate AD's progression and cure AD effectively.

**Figure 11:**
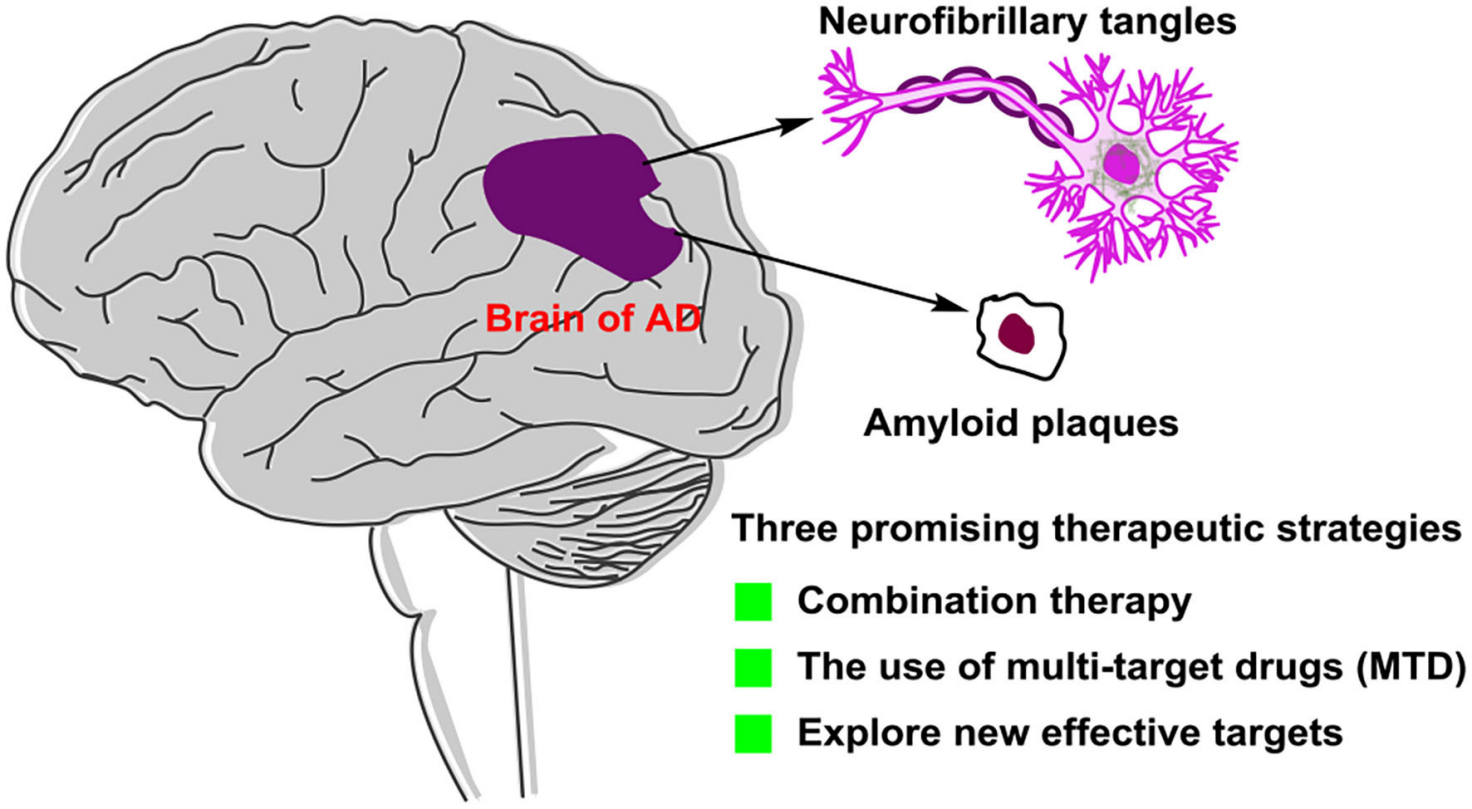
Three promising therapeutic strategies for Alzheimer's disease (AD) in future.

The failure of clinical trials, which were based on the generally accepted hypothesis, encouraged researchers to consider new therapeutic agents and strategies with mechanisms independent of traditional hypothesis. To conquer this complicated disease, we think that three strategies might be attractive directions in future (Figure 11). The first is combination therapy. Combination of multiple small-molecule target-specific drugs that targeted different critical targets or combination of one small-molecule drug with antibodies may give additive effects in AD treatment. The second is the use of multitarget drugs (MTDs). The development of an MTD approach is based on combination therapy and shows improved pharmacokinetics, safety, and patient compliance. However, great efforts at improving MTD's pharmacodynamics and efficacy should be made. The third is in discovering new effective targets. Understanding the pathology of AD intensively and discovering new targets is one of the potential aspects considered in AD treatment. Designing new drugs targeted new effective targets may open up a new way for AD treatment.

This review summarizes some representative AD-treatment small-molecule drugs that entered clinical trials mostly, alleviating the symptoms of AD through the development of cholinergic inhibitors, Aβ inhibitors, tau protein inhibitors, and other targeted drugs. We hope this review can inspire the research enthusiasm of researchers to advance the discovery and development of new-generation drugs for AD in future, which will conquer and cure this disease finally.

## Author contributions

WY was responsible for literature research and drafting of manuscripts. HY participated in literature research and sorting. JY was responsible for the revision, improvement, and submission of manuscripts. All authors contributed to the article and approved the submitted version.

## Funding

This study was supported by the Taishan Scholars Project of Shandong Province (tsqn202103108 to JY).

## Conflict of interest

The authors declare that the research was conducted in the absence of any commercial or financial relationships that could be construed as a potential conflict of interest.

## Publisher's note

All claims expressed in this article are solely those of the authors and do not necessarily represent those of their affiliated organizations, or those of the publisher, the editors and the reviewers. Any product that may be evaluated in this article, or claim that may be made by its manufacturer, is not guaranteed or endorsed by the publisher.
